# Comprehensive Analysis of the Role of *SLC2A3* on Prognosis and Immune Infiltration in Head and Neck Squamous Cell Carcinoma

**DOI:** 10.1155/2022/2371057

**Published:** 2022-09-19

**Authors:** Manru Chu, Ke Zheng, Xiaojie Li, Zhiqiang Luo, Xin Yang, Changbo Wei

**Affiliations:** ^1^Suzhou Stomatological Hospital, Suzhou 215000, China; ^2^Department of Stomatology, Wuxi No.2 People's Hospital, Wuxi 214000, China; ^3^Genetics and Cellular Engineering Group, Institute of Molecular Toxicology and Pharmacology, Helmholtz Zentrum Munich, Neuherberg 85764, Germany

## Abstract

**Background:**

*SLC2A3* is upregulated in various cancer types and promotes proliferation, invasion, and metabolism. However, its role in the prognosis and immune regulation of head and neck squamous cell carcinoma (HNSCC) is still obscure. This study is aimed at exploring the prognostic and immunotherapeutic potential of *SLC2A3* in HNSCC.

**Methods:**

All data were downloaded from TCGA database and integrated via R software. *SLC2A3* expression was evaluated using R software, TIMER, CPTAC, and HPA databases. The association between *SLC2A3* expression and clinicopathologic characteristics was assessed by R software. The effect of *SLC2A3* on survival was analyzed by R software and Kaplan-Meier Plotter. Genomic alterations in *SLC2A3* were investigated using the cBioPortal database. Coexpression of *SLC2A3* was studied using LinkedOmics and STRING, and enrichment analyses were performed with R software. The relationship between *SLC2A3* expression and immune infiltration was determined using TIMER and TISIDB databases. Immune checkpoints and ESTIMATE score were analyzed via the SangerBox database.

**Results:**

*SLC2A3* expression was upregulated in HNSCC tissues compared to normal tissues. It was significantly related to TNM stage, histological grade, and alcohol history. High *SLC2A3* expression was associated with poor prognosis in HNSCC. Coexpression analysis indicated that *SLC2A3* mostly participated in the HIF-1 signaling pathway and glycolysis. Furthermore, *SLC2A3* expression strongly correlated with tumor-infiltrating lymphocytes in HNSCC.

**Conclusion:**

*SLC2A3* could serve as a potential prognostic biomarker for tumor immune infiltration in HNSCC.

## 1. Introduction

Head and neck squamous cell carcinoma (HNSCC) is one of the top 10 cancer types worldwide and has a high mortality rate [1]. Despite advances in comprehensive cancer therapies, including traditional surgery, radiotherapy, and chemotherapy, the 5-year survival rate for HNSCC remains around 50%; this substantially increases the burden on family and society because most patients are middle-aged adults [2, 3]. The high mortality due to HNSCC accounts for the aggressive proliferation resulting in high recurrence and metastasis. The lack of early screening and diagnostic biomarkers is responsible for the low survival rate, while most patients with metastatic HNSCC die within 1 year after diagnosis [4].

Recently, many endeavors have been made to explore the biomarkers for more effective treatments for HNSCC [4–6]. Therapeutic advances in immunotherapy have revolutionized as promising methods for cancer treatment, and a small number of patients have benefited from them. The tumor immune microenvironment (TIME) is a dynamic structure comprising complex components, in which the immune system can influence the growth and mutation of tumor cells by yielding antitumor immune responses [7]. At the same time, tumor cells also damage immune cells differently. HNSCC is one of the most immunosuppressive human cancer types, so immune-based therapy could effectively prolong patients' survival by preventing tumor immune escape and stimulating antitumor immune responses. Thus, it is imperative to explore novel noninvasive biomarkers for improving the prognosis and immunotherapy in HNSCC.

*SLC2A3*, also annotated as GLUT3, is a member of the solute carrier 2A (SLC2A) family participating in glucose transport across the hydrophobic cell membrane in mammalian cells. Cancer cells mainly rely on glycolysis for nutrient intake and energy production [8]. There is a growing body of literature that focusing on the upregulating of *SLC2A3* to research cancer diagnosis and therapy [9]. In colorectal cancer, *SLC2A3* plays a crucial role in perineural invasion via the regulation of epithelial-mesenchymal transition [10]. *SLC2A3* could predict poor prognosis in gastric cancer, associated with macrophage infiltration in the tumor microenvironment (TME) [11]. Similarly, *SLC2A3* overexpression in triple-negative breast cancer could activate inflammatory M1 tumor-associated macrophages resulting in the aggressiveness of tumor cells [12]. With the deep interrogation of glycolysis-related gene *SLC2A3* in diverse cancer types, there is a pressing need to research the correlation between *SLC2A3* and immune infiltration. However, the relationship between *SLC2A3* and HNSCC remains unclear.

In the present study, we identified the differential expression levels of *SLC2A3* gene and protein between tumor tissues and normal tissues based on The Cancer Genome Atlas (TCGA) and several online databases. The interaction networks of *SLC2A3* and enrichment analyses were performed. Prognostic value and clinicopathologic features of *SLC2A3* in HNSCC were evaluated. We conducted immune infiltration analyses to explore the potential immune mechanisms of *SLC2A3* in HNSCC. Our study suggests that *SLC2A3* could act as a valuable prognostic biomarker. The study also sheds light on the association of *SLC2A3* with immune infiltration in HNSCC.

## 2. Materials and Methods

### 2.1. Data Extraction

The transcriptome profiling of *SLC2A3* and related clinical information was extracted from TCGA database (https://portal.gdc.cancer.gov/). The RNA-seq gene expression with the format of FPKM was transformed into TPM format, and log2 conversion was conducted for further analyses. Gene expression data of *SLC2A3* in HNSCC were acquired from TCGA and involved 502 tumor tissue and 44 adjacent normal tissue data. According to the median expression level of *SLC2A3*, gene expression data were divided into two subgroups: the high expression group and the low expression group. The samples contained several clinical characteristics, including TNM stage, clinical stage, histologic grade, gender, age, smoke condition, alcohol history, radiation therapy, primary therapy outcome, and lymphovascular invasion (Table 1). The RNA expression data were performed by mean ± SD using R software (version 3.6.3). All the data were downloaded from public databases; therefore, no ethical approval document was required.

### 2.2. Gene Expression of *SLC2A3* in Human Cancers and HNSCC

The gene expression of *SLC2A3* in human cancers/HNSCC and paired normal tissues were analyzed using the “ggplot2” package. Tumor Immune Estimation Resource (TIMER) is a user-friendly online web resource for the systematic analysis of immune infiltrates in different cancers (https://cistrome.Shinyapps.io/timer/). We used the TIMER database to verify the differential expression of *SLC2A3* in pan-cancer [13].

### 2.3. Protein Expression of *SLC2A3*

UALCAN is a comprehensive web resource referring to publicly available cancer omics data (http://ualcan.path.uab.edu). Clinical Proteomic Tumor Analysis Consortium (CPTAC) is focused on molecular mechanisms of cancer via the application of proteome and genome analysis or proteogenomics (https://proteomics.cancer.gov/programs/cptac) [14]. The Human Protein Atlas (HPA) is aimed at exploring all the human proteins in cells, tissues, and organs, and all the data were freely available to the public for research on the human proteome (https://proteinatlas.org/) [15]. In the present study, UALCAN was used to analyze the protein expression of *SLC2A3* from CPTAC, and HPA was performed to analyze the protein expression of *SLC2A3* in normal tissue and tumor tissue of HNSCC.

### 2.4. Correlation between Clinical Pathological Characteristics and *SLC2A3*

To explore the association between *SLC2A3* expression and clinical pathological characteristics including TNM stage, clinical stage, gender, age, histologic grade, smoker condition, and alcohol history, we applied the “ggplot2” package based on the TCGA database. The statistical significance was set at *P* < 0.05.

### 2.5. The Prognostic Value of *SLC2A3*

The ROC curve was drawn to examine the cutoff value of *SLC2A3* using the “pROC” and “ggplot2” packages. The overall survival (OS) and disease-specific survival (DSS) between the high expression group and the low expression group were evaluated using the “survival” package and visualized using the “survminer” package. *P* < 0.05 was regarded as statistically significant. The Kaplan-Meier Plotter collected discovery and validation of survival biomarkers to analyze the correlations between gene expression and survival in multiple tumor types (http://kmplot.com/analysis/) [16]. According to the Kaplan-Meier Plotter database, we explored the relapse-free survival (RFS) associated with *SLC2A3* expression levels in HNSCC. The prognostic value was statistically significant when the *P* < 0.05.

### 2.6. Construction and Evaluation of Nomogram

To predict 1-, 3-, and 5-year survival probability associated with *SLC2A3* in HNSCC, we applied the “RMS” package to construct a predictive model of nomogram including several clinical characteristics. Calibration and discrimination analysis is a very effective predictive model evaluation method. In this study, variables of radiation therapy, primary therapy outcome, lymphovascular invasion, and *SLC2A3* expression were brought in to map the nomogram-predicted probabilities. The 45-degree line in calibration curves represented the best predictive value. The C-index and ROC curves were used to examine the predictive accuracies of the nomogram and individual prognostic factors. The statistical significance was set at *P* < 0.05.

### 2.7. Genomic Alterations of *SLC2A3* in HNSCC

cBio Cancer Genomics Portal (cBioPortal) is an online website for analyzing multidimensional cancer genomics data (http://cbioportal.org). Thus, cBioPortal was used to investigate the genomic alterations in HNSCC [17].

### 2.8. Coexpression and Enrichment Analysis of *SLC2A3*

The LinkedOmics database provides a public platform to analyze cancer multiomics data from all 32 TCGA cancer types and 10 CTPCA cancer cohorts (http://www.linkedomics.org/login.php). Thus, we applied LinkedOmics to examine the coexpression of *SLC2A3* with Pearson's correlation coefficient [18]. Search Tool for the Retrieval of Interacting Genes/Proteins (STRING) is a database for computational predicted physical and functional protein-protein interactions (http://string-db.org, version 11.5). In this study, STRING analysis was conducted to explore the coexpression genes associated with *SLC2A3* [19]. The network was built, and the minimum required interaction score was set at 0.7 (high confidence). Functional enrichment analyses were performed using the “ggplot2” and “ClusterProfiler” packages. Briefly, Gene Oncology (GO) was annotated, and the Kyoto Encyclopedia of Genes and Genomes (KEGG) was analyzed to illustrate the potential mechanisms.

### 2.9. Correlation between *SLC2A3* Expression and Immune Infiltration Cells

We applied TIMER to investigate the association between immune infiltrates (B cell, CD8^+^ T cell, CD4^+^ T cell, macrophage, neutrophil, and dendritic cell) and *SLC2A3* expression or clinical outcome in HNSCC. Tumor Immune System Interaction Database (TISIDB) integrates diverse data types for exploring the interaction between tumors and the immune system (https://cis.hku.hk/TISIDB) [20]. In this study, we performed TISIDB to examine the correlation between *SLC2A3* expression and the abundance of 28 tumor-infiltrating lymphocytes (TILs) acquired by gene set variation analysis. The association between immune or molecular subtypes and *SLC2A3* expression in HNSCC was also explored via TISIDB. The difference at a *P* < 0.05 was regarded as statistically significant.

### 2.10. Relationship between *SLC2A3* Expression and Immune Checkpoint (ICP) Genes, and ESTIMATE Score of TME in HNSCC

SangerBox website (http://sangerbox.com/Tool) was used to investigate the correlation between *SLC2A3* expression and immune checkpoint (ICP) genes, and ESTIMATE (Estimation of STromal and Immune cells in MAlignant Tumor tissues using Expression data) score based on TCGA and GTEx databases. 60 ICP genes were analyzed in this study. ESTIMATE is an algorithm to predict levels of infiltrating stroma and immune cells by calculating stroma and immune scores, further deducing tumor purity [21]. Differences with a *P* < 0.05 were regarded to be statistically significant.

### 2.11. Statistical Analyses

All statistical analyses were performed by R software (version 3.6.3). Mann–Whitney *U* test and Wilcoxon signed rank test were used to compare *SLC2A3* expression levels in normal tissues and tumor tissues. The Wilcoxon rank sum test was performed to evaluate the relationships between clinical-pathologic characteristics. The “pROC” package was used to examine the cutoff value of *SLC2A3* in the ROC curve. The log-rank test and Cox regression analyses were used to evaluate the OS.

## 3. Results

### 3.1. The Differential Expression of *SLC2A3* in Pan-Cancer

**Figure 1:**
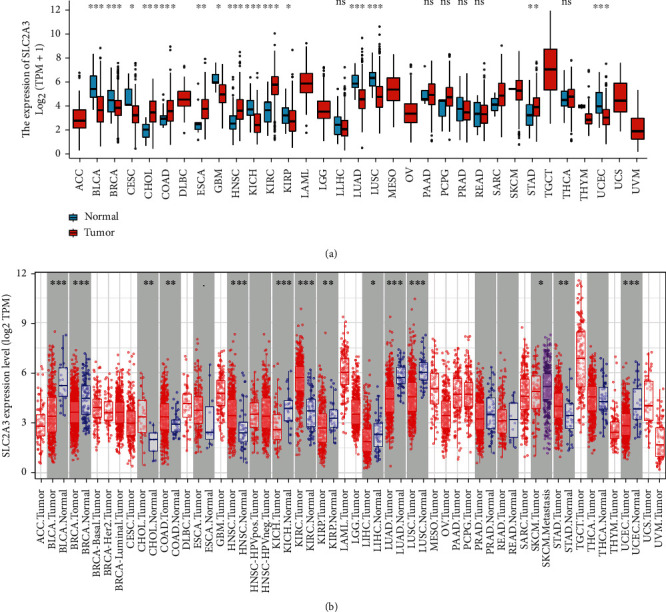
*SLC2A3* expression levels in human cancers determined using R software (a) and TIMER database (b). (ns, no significance, ^∗^*P* < 0.05, ^∗∗^*P* < 0.01, ^∗∗∗^*P* < 0.001).

Analysis of the mRNA expression of *SLC2A3* in 33 different types of cancers showed that *SLC2A3* was upregulated in six cancer types, including cholangiocarcinoma (CHOL), colon adenocarcinoma (COAD), esophageal carcinoma (ESCA), head and neck carcinoma (HNSC), kidney renal clear cell carcinoma (KIRC), and stomach adenocarcinoma (STAD) compared to normal tissues (Figure 1(a)). However, *SLC2A3* expression levels were significantly lower in nine types of cancer, including bladder urothelial carcinoma (BLCA), breast invasive carcinoma (BRCA), cervical and endocervical cancer (CESC), glioblastoma multiforme (GBM), kidney chromophobe (KICH), kidney renal papillary carcinoma (KIRP), lung adenocarcinoma (LUAD), lung squamous cell carcinoma (LUSC) and uterine corpus endometrial carcinoma (UCEC). As displayed in Figure 1(b), the results of the TIMER database were similar to those obtained by R software. The *SLC2A3* expression level was significantly higher in CHOL, COAD, ESCA, HNSC, KIRC, and STAD than in normal tissue. However, the *SLC2A3* expression level was lower in BCLA, BRCA, KICH, KIRP, liver hepatocellular carcinoma (LIHC), LUAD, LUSC, and UCEC.

### 3.2. mRNA and Protein Overexpression of *SLC2A3* in HNSCC

**Figure 2:**
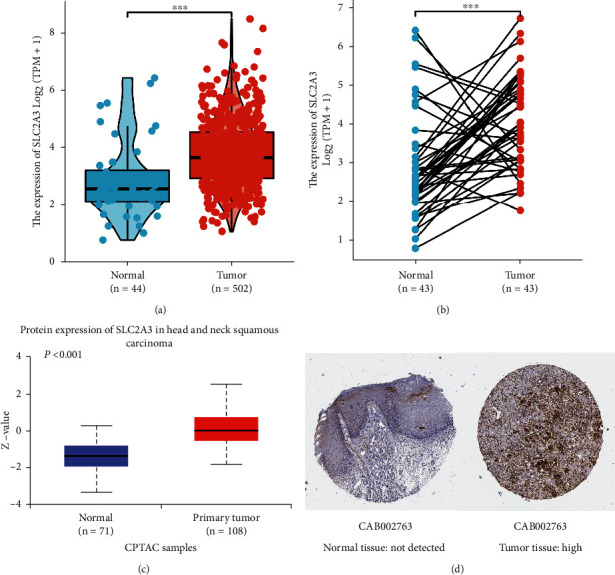
Gene expression levels of *SLC2A3* in normal tissues and tumor tissues by unpaired analysis (a) and paired analysis (b). Protein expression levels of *SLC2A3* in normal tissues and tumor tissues based on CPTAC (c) and HPA (d). (∗∗∗*P* < 0.001).

Unpaired data analyses showed that the mRNA expression levels of *SLC2A3* in 502 HNSCC tissues were significantly higher than those in 44 normal tissues (*P* < 0.001; Figure 2(a)). In the paired samples, the results were consistent with the above analyses (Figure 2(b)). *SLC2A3* was significantly upregulated in 43 tumor tissues than in paired normal tissues (*P* < 0.001). To explore the protein expression of *SLC2A3*, we performed analysis from HPA and CPTAC with UALCAN. The results revealed that the protein of *SLC2A3* was overexpressed in HNSCC compared to normal tissues (Figures 2(c) and 2(d)).

### 3.3. Expression of *SLC2A3* and Different Clinicopathologic Variables

**Figure 3:**
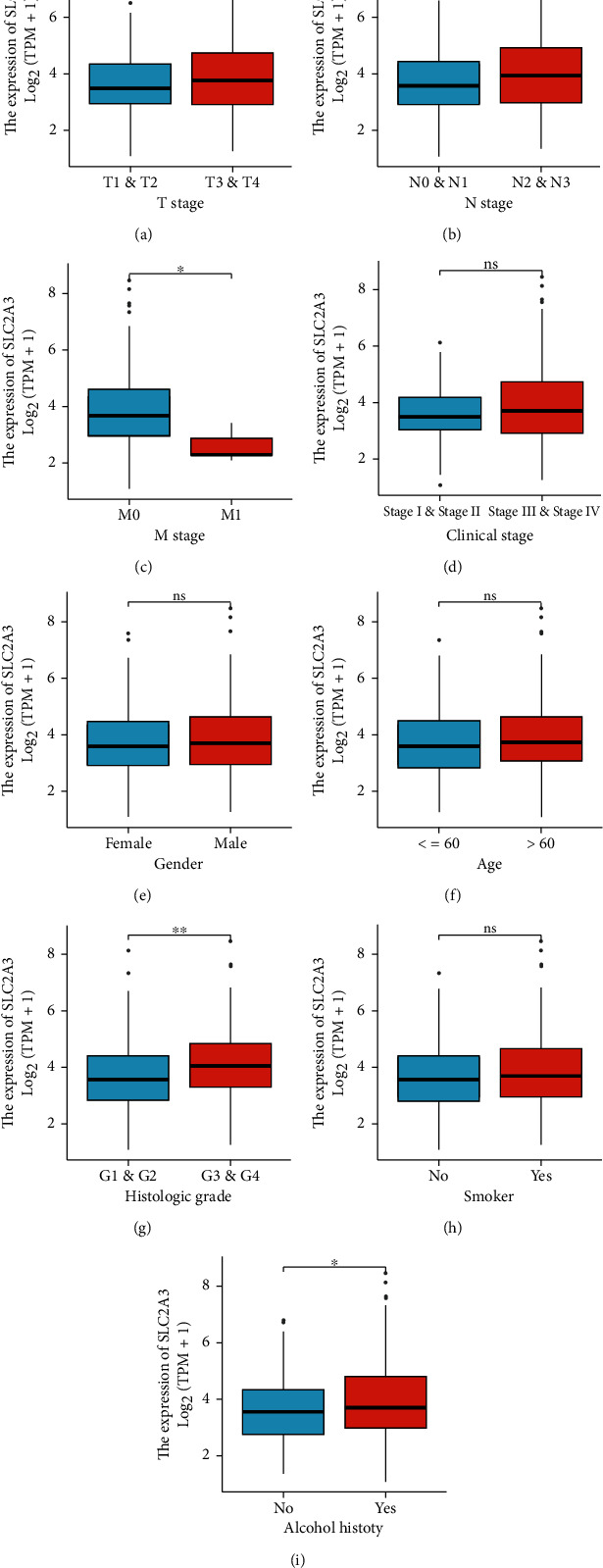
Correlation of *SLC2A3* expression with different clinicopathologic variables including T stage (a), N stage (b), M stage (c), clinical stage (d), gender (e), age (f), histologic grade (g), smoker condition (h), and alcohol history (i). (ns, no significance, ^∗^*P* < 0.05, ^∗∗^*P* < 0.01).

The Wilcoxon rank sum test showed that the expression level of *SLC2A3* was significantly related to T stage (*P* = 0.045), N stage (*P* = 0.004), M stage (*P* = 0.012), histological grade (*P* = 0.002), and alcohol history (*P* = 0.015). However, no significant correlations were detected between *SLC2A3* expression and other clinicopathologic variables, such as clinical stage (*P* = 0.071), gender (*P* = 0.501), age (*P* = 0.076), and smoker condition (*P* = 0.223) (Figure 3).

### 3.4. High Expression of *SLC2A3* Related to Poor Prognosis of HNSCC

**Figure 4:**
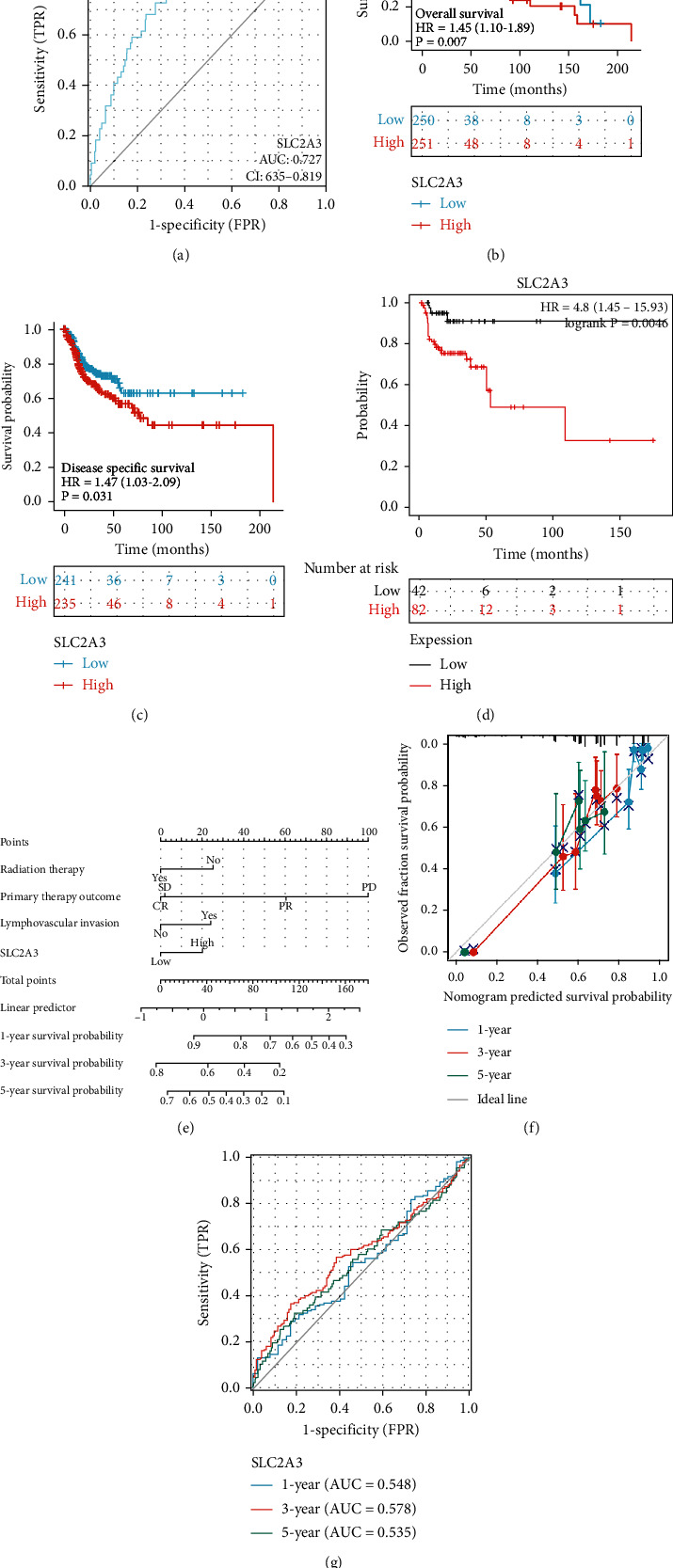
Effect of *SLC2A3* on HNSCC prognosis. ROC curve showed promising predictive power (a). High expression of *SLC2A3* related to poor OS and DSS, respectively, analyzed using R software (b, c). High expression of *SLC2A3* related to RFS, analyzed using Kaplan-Meier Plotter database (d). A nomogram for predicting 1-, 3-, and 5-year survival probability (e). ROC curve for 1-, 3-, and 5-year OS showed the AUG values of 0.548, 0.578, and 0.535, respectively (f). Calibration plot of the nomogram-predicted survival (g).

Several methods were applied to analyze the prognosis associated with *SLC2A3* expression in HNSCC. Firstly, the ROC curve showed promising predictive power with an AUG value of 0.727 (95% CI: 0.635-0.819; Figure 4(a)). Secondly, the results from “survival” and “survminer” packages showed that higher expression level of *SLC2A3* was significantly related to poorer OS (HR = 1.45, *P* = 0.007; Figure 4(b)) and DSS (HR = 1.47, *P* = 0.031; Figure 4(c)). Meanwhile, analyses from the Kaplan-Meier Plotter database corresponded with the above results. Overexpression of *SLC2A3* was correlated with RFS (HR = 4.8, *P* = 0.0046; Figure 4(d)). Lastly, a nomogram was constructed to analyze the survival independently associated with several clinical characteristics, including radiation therapy, primary therapy outcome, lymphovascular invasion, and *SLC2A3* expression (Figure 4(e)). The points scale was used to distribute every single variable, and the total points scale represented the sum of a point from every single variable. The survival probability at 1, 3, and 5 years corresponded to the total points. Thus, *SLC2A3* was a poor prognostic factor for OS in HNSCC. The C-index of the nomogram was 0.714 (95% CI: 0.687-0.741), and the time-dependent ROC curve to 1-, 3-, and 5-year OS showed the AUG value of 0.548, 0.578, and 0.535, respectively (Figure 4(f)). In addition, the bias-corrected line in the calibration was close to the 45-degree line (Figure 4(g)).

### 3.5. Genomic Alterations of *SLC2A3* in Six Data Sets

**Figure 5:**
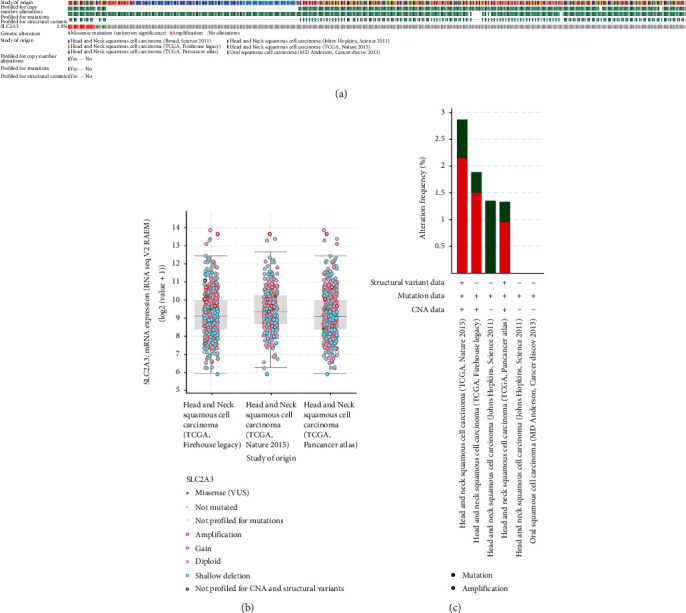
Genomic alterations of *SLC2A3* in six data-sets investigated using the cBioPortal database. OncoPrint of *SLC2A3* expression in HNSCC (a). *SLC2A3* gene alteration types in HNSCC (b). Details of *SLC2A3* gene alteration types in HNSCC (c).

The exploration of genomic alterations of *SLC2A3* in six HNSCC data sets was conducted with the cBioPortal website. Genomic alteration of *SLC2A3* occurred in 1.8% of patients with HNSCC (Figure 5(a)). The gene alteration type was diverse associated with *SLC2A3* mRNA expression (Figure 5(b)). Copy number variation (CNV) mainly occurred in HNSCC. While narrowing the cancer field, no CNV was detected in oral squamous cell carcinoma (OSCC) (Figure 5(c)).

### 3.6. *SLC2A3* Coexpression Networks and Enrichment Analyses

**Figure 6:**
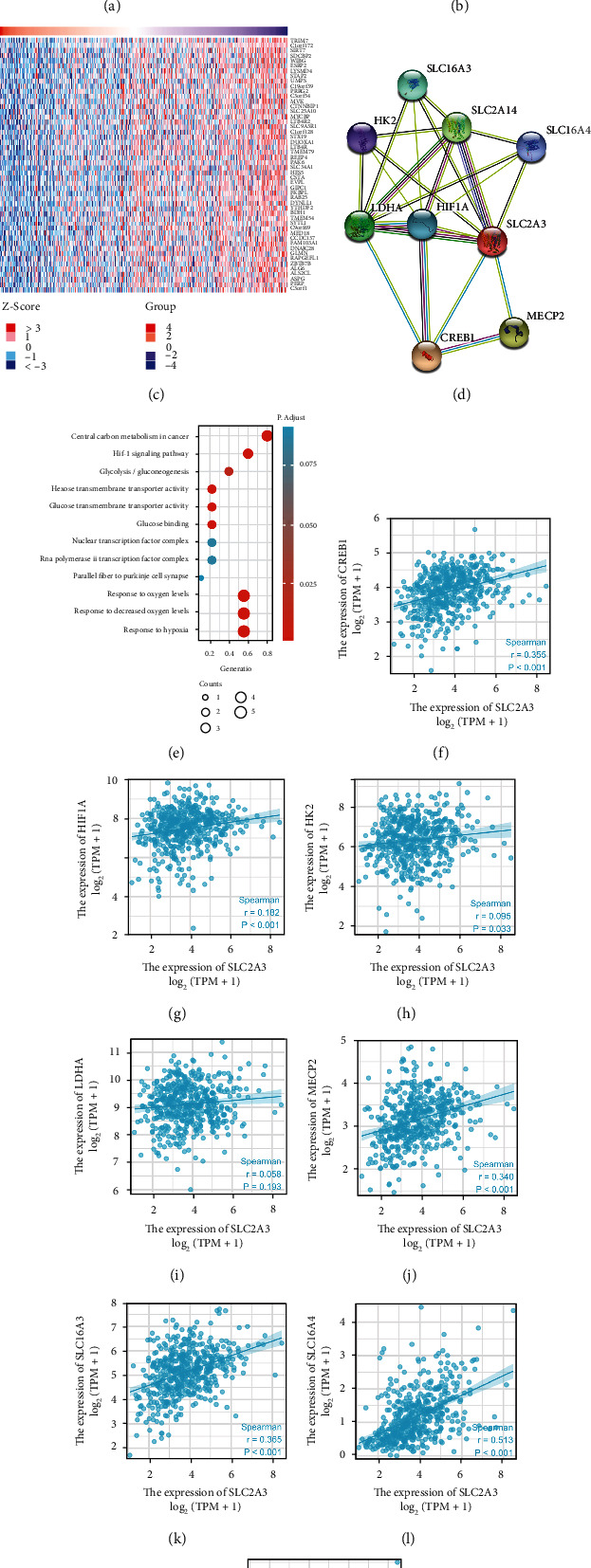
*SLC2A3* coexpression networks and enrichment analyses. Coexpression genes of *SLC2A3* analyzed by Pearson test based on LinkedOmics database (a). Top 50 positive coexpression genes (b). Top 50 negative coexpression genes (c). PPI networks associated with *SLC2A3* (d). Enrichment analysis of nine coexpression genes (e). Correlation analyses between *SLC2A3* and coexpression genes (f–m).

With the LinkedOmics database, coexpression genes of *SLC2A3* were displayed as volcano plots in which 7089 genes were significantly positively correlated to *SLC2A3*, and 4057 genes were negatively related to *SLC2A3* (Figure 6(a)). The heat maps showed the top 50 genes positively and negatively associated with *SLC2A3*, respectively (Figures 6(b) and 6(c)).  Figure 6(d) shows a PPI network of *SLC2A3* and its eight coexpression genes. We conducted “clusterProfiler” and “ggplot2” with these eight genes to explore the GO and KEGG enrichment. Coexpression genes were mainly involved in several key functions, including biological processes of response to oxygen levels, response to decreased oxygen levels, and response to hypoxia; cellular components of nuclear transcription factor complex, RNA polymerase II transcription factor complex, and parallel fiber to Purkinje cell synapse; and molecular functions of hexose transmembrane transporter activity, glucose transmembrane transporter activity, and glucose binding (Figure 6(a)). At the same time, KEGG results showed that coexpression genes enriched in central carbon metabolism in cancer, the HIF-1 signaling pathway, and glycolysis/gluconeogenesis. In addition, the correlation analyses between coexpression genes and *SLC2A3* in HNSCC are displayed in Figure 6(f)–6(m).

### 3.7. Correlation between *SLC2A3* Expression and Immune Cell Infiltration

**Figure 7:**
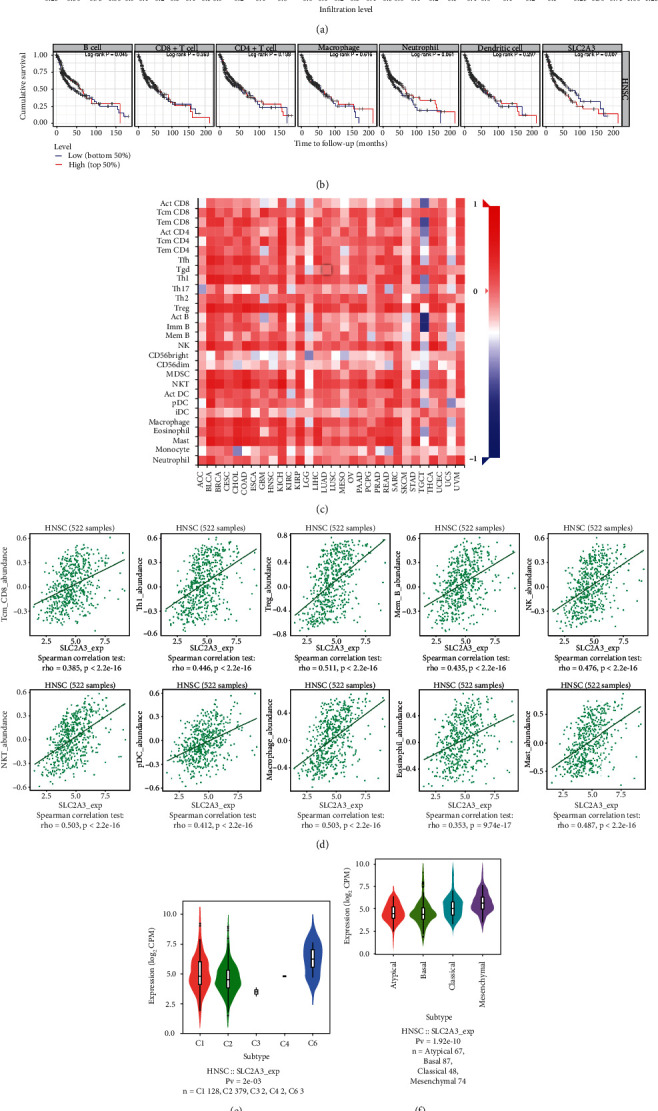
Correlation between *SLC2A3* expression and immune cell infiltration. *SLC2A3* expression was significantly related to the abundances of B cells, CD8^+^ T cells, CD4^+^ T cells, macrophages, neutrophils, and dendritic cells (a). Cumulative survival was poor significantly associated with the abundance of B cells (b). Correlations between expression of *SLC2A3* and 28 TIL types across human cancers, investigated using the TISIDB database (c). *SLC2A3* was significantly correlated with abundances of Tcm CD8 cells, Th1 cells, Treg cells, Mem B cells, NK cells, NKT cells, pDCs, macrophages, eosinophils, and mast cells (d). Relationship between *SLC2A3* expression and immune subtypes (e). Relationship between *SLC2A3* expression and molecular subtypes (f).

The correlation between *SLC2A3* expression and six immune cells was analyzed using the TIMER database (Figure 7(a)). In HNSCC, *SLC2A3* expression level was significantly related to B cell (*r* = 0.222, *P* = 9.96e − 07), CD8^+^ T cell (*r* = 0.229, *P* = 4.75e − 07), CD4^+^ T cell (*r* = 0.372, *P* = 3.45e − 17), macrophage (*r* = 0.516, *P* = 3.63e − 34), neutrophil (*r* = 0.322, *P* = 4.96e − 13), and dendritic cell (*r* = 0.49, *P* = 1.58e − 30). The cumulative survival was significantly associated with the abundance of B cells according to Figure 7(b). While the correlations between the expression levels of *SLC2A3* and 28 TIL types across human cancers were explored in the TISIDB database (Figure 7(c)). Twenty-two types of TILs significantly related to *SLC2A3* expression levels in HNSCC, such as central memory CD8 T (Tcm CD8) cells, type 1 helper (Th1) cells, regulatory T (Treg) cells, memory B (Mem B) cells, natural killer (NK) cells, natural killer T (NKT) cells, plasmacytoid dendritic cells (pDCs), macrophages, eosinophils, and mast cells (mast) (Figure 7(d)). Additionally, the role of *SLC2A3* on immune and molecular subtypes in HNSCC was analyzed (Figures 7(e) and 7(f)). Immune subtypes were divided into six types: C1 (wound healing), C2 (IFN-gamma dominant), C3 (inflammatory), C4 (lymphocyte depleted), C5 (immunologically quiet), and C6 (TGF-b dominant). *SLC2A3* showed high expression in the C6 type and low expression in C1 and C2 types. The molecular subtypes of cancers were classified into four types: atypical, basal, classical, and mesenchymal. *SLC2A3* was upregulated in classical and mesenchymal types and downregulated in atypical and basal types. For different immune and molecular subtypes, *SLC2A3* expression was significantly different in HNSCC.

### 3.8. *SLC2A3* Expression Is Related to Immune Checkpoint (ICP) Genes and ESTIMATE Score in HNSCC

**Figure 8:**
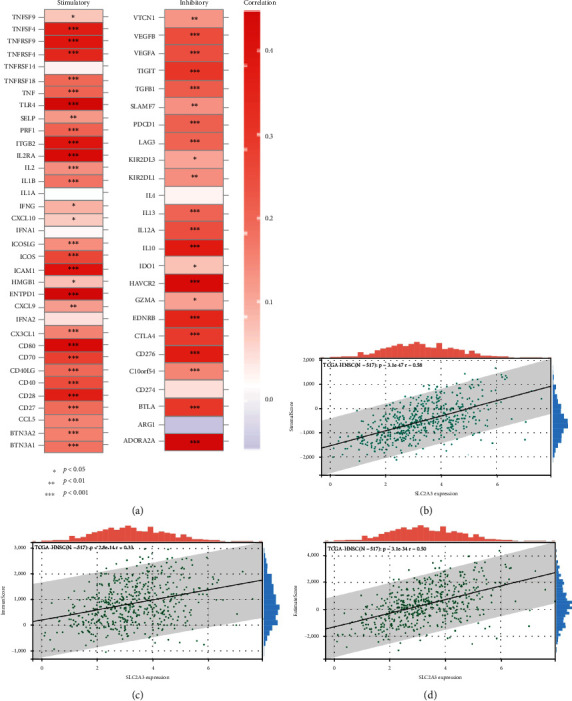
Relationship of *SLC2A3* expression with ICP genes and ESTIMATE score in HNSCC. Of 60 ICP genes, 53 significantly correlated with *SLC2A3* expression in HNSCC (a). Significantly positive relationships were noted with stromal score (*P* = 3.1e − 47), immune score (*P* = 2.8e − 14), and ESTIMATE score (*P* = 3.1e − 34) (b).

We found that 53 of 60 ICP genes significantly correlated to *SLC2A3* expression in HNSCC, in which 21 genes were inhibitors and 32 were stimulators (Figure 8(a)). The associations between *SLC2A3* expression and three kinds of ESTIMATE score (Figure 8(b)–8(d)). The results indicated significant positive relationships with stromal score (*P* = 3.1e − 47), immune score (*P* = 2.8e − 14), and ESTIMATE score (*P* = 3.1e − 34) in HNSCC.

## 4. Discussion

The SLC2A family comprises 14 members from SLC2A1-SLC2A14, among which those genes exhibit a tissue-specific expression. For instance, SCL2A1 is widely expressed in most tissues, while SLC2A2 is predominantly expressed in liver tissues [22]. *SLC2A3* was mainly expressed in the central nervous system, which relies on steady support for ATP generation [23]. However, overexpression of *SLC2A3* was found in several cancers such as colorectal cancer, gastric cancer and breast cancer [10–12]. In acute myeloid leukemia (AML), a *SLC2A3*-knockdown cell line accounted for the less effective demethylation and vitamin C in the AML and lymphoma cell lines suggesting *SLC2A3* could act as a predictive biomarker in vitamin C treatment in AML [24]. In GBM, *SLC2A3* played a vital oncogenic role in attenuating cell proliferation and glucose uptake via miR-106a [25]. Thus, According to the mediation in glucose transport, *SLC2A3* was regarded as a cancer-promoting gene. The prognostic role of *SLC2A3* in cancers has recently gained attention. High *SLC2A3* expression resulted in shorter OS and disease-free survival in colorectal carcinoma (CRC) [26]. We performed bioinformatics to study the correlation between *SLC2A3* and prognosis in HNSCC based on transcriptome profiling using the TCGA database and several online websites. It was found that *SLC2A3* is differentially expressed in many cancer types, which was consistent with the above results. Moreover, upregulation of *SLC2A3* in HNSCC was demonstrated, and high expression of *SLC2A3* was significantly related to TNM stage, histologic grade, and alcohol history, suggesting *SLC2A3* functioned as an oncogene in HNSCC. A previous study revealed that siRNA-mediated silencing of *SLC2A3* could suppress the growth and promote apoptosis in AML cells [27]. Further, high expression of *SLC2A3* was associated with poor OS, DSS, and RFS compared to low expression. Similarly, the immunohistochemical analysis of *SLC2A3* was conducted in OSCC tissues, and the results showed that positive expression of *SLC2A3* was correlated to unfavorable prognosis outcomes [28]. We concluded that *SLC2A3* could act as a biomarker for prognosis in HNSCC.

Many studies have demonstrated that *SLC2A3* could serve as an oncogene because of the synergistic action with upstream and downstream molecules. In GBM, knockdown of HDAC2 could induce GBM cell death by miR-3189-targeted *SLC2A3* repression through reprogramming of glucose metabolism [29]. YAP played a crucial role in promoting the proliferation and migration of CRC cells by activating the *SLC2A3*/AMPK signaling pathway and epithelial-to-mesenchymal transition (EMT) [30]. Other studies have shown that *SLC2A3* was directly related to SOX9, TRIM66, and HMGA1, which could promote cancer cell proliferation, migration, and invasion [31–33]. Given the condition, we constructed the coexpression network and performed the enrichment analysis for *SLC2A3* in HNSCC. Interestingly, it was found that *SLC2A3* was significantly correlated to glycolysis-related gene HK2 and hypoxia-related gene HIF1A, which indicated that the expression of *SLC2A3* in HNSCC was regulated via glycolysis and HIF-1 signaling pathway according to enrichment analysis. In prostate cancer, *SLC2A3* was a direct target of miR-29c to inhibit cell growth and glucose metabolism [34]. Similar results showed that miR-129-5p directly targets *SLC2A3* to suppress glucose consumption, lactate generation, cellular ATP levels, and glucose uptake in gastric cancer cells [35]. Furthermore, immune cells use the ATPs generated by increasing glycolysis to modulate functional activities, growth, volume, and ion integrity, and antiglycolytics limit immunity [36]. However, research based on bioinformatics showed that high glycolysis status significantly correlated with less immune function, further related to poor prognosis in patients with osteosarcoma [37]. Hypoxia is a common feature in the TME because the uncontrolled proliferation of tumor cells mismatches the relatively insufficient supply of blood as well as oxygen, leading to stress-tolerant tumor cell survival [38]. In hypoxic-cultured prostate cell lines and hypoxic regions of xenograft tumors, the expression of *SLC2A3* was significantly increased [39]. Combining previous results with our present study, we speculate that *SLC2A3* predicting unfavorable prognosis in HNSCC could associate with glucose metabolism and HIF-1 signaling pathway.

It has long been acknowledged that the comprehensive immunobiology of the TME is modulated by several factors, including TILs, location of the tumor, and tumor stromal and vasculature [40–44]. The relationship between *SLC2A3* and immune infiltration in human cancers is not widely studied. A recent study observed that *SLC2A3* was one of an-eight gene prognostic risk score model to predict the survival significantly related to immune cell infiltration [45]. In this study, we found that *SLC2A3* strongly correlated to TILs in HNSCC by TIMER, such as B cell, CD8^+^ T cell, CD4^+^ T cell, macrophage, neutrophil, and dendritic cell. For example, macrophages have been proven to account for immune evasion and suppression, and CD8^+^ T cells could mediate the recognition of the antigens expressed by most tumor cells [46]. Interestingly, B cell was significantly associated with the cumulative survival in HNSCC between high expression and low expression of *SLC2A3* suggesting that *SLC2A3* could serve as an immune-related prognostic biomarker. We also demonstrated a positive correlation between *SLC2A3* expression and Tcm CD8, Th1, Treg, Mem B, NK, NKT, pDC, eosinophil, and mast cells. In prostate cancer, *SLC2A3* was one of the high-risk genes, and the risk score was significantly related to NK cell infiltration [47]. While functional Treg was generated from naive T cells lacking Glut1, despite expressing a low level of *SLC2A3* which mediates glucose uptake [48]. Then, the expression of *SLC2A3* was explored, and the results showed significantly differential expression of *SLC2A3* in different immune and molecular subtypes. A previous study demonstrated that glucose metabolism could be applied in conjunction with several inflammatory diseases like rheumatoid arthritis, in which rheumatoid arthritis fibroblast-like synoviocytes were cultured with CD4 T cells or T cell conditioned medium. Then it was observed that the secretion of inflammatory genes including TNF-*α*, IFN-*γ*, IL-17A, IL-6, and IL-8 was augmented, and glucose transporters like GLUT1 and *SLC2A3* were increased. [49]. According to these results, we hypothesized that *SLC2A3* could play an essential role in HNSCC immune microenvironment. Moreover, ICP genes are important immune regulators in maintaining self-tolerance and modulating the immune response by both stimulatory and inhibitory pathways [50]. T cell-associated inhibitory molecules like lymphocyte activation gene 3 (LAG3) could suppress the antitumor immune responses, while upregulation of inducible costimulator could contribute to favorable clinical outcomes [51]. When it comes to *SLC2A3*, a recent study observed that *SLC2A3* knockdown significantly inhibited the expression of PD-L1 indicating *SLC2A3* could participate in the immune response by regulating the PD-L1 immune checkpoint in CRC [10]. Similarly, upregulation of *SLC2A3* could enhance CD28 costimulation reprogrammed renal cell carcinoma CD8^+^ TIL metabolism, which is antagonized by the inhibitory and checkpoint immunotherapy receptors CTLA4 and PD-1 [52]. We found a strong relationship between *SLC2A3* and ICP genes which provided a novel insight for targeted immunotherapy in HNSCC. In addition, we found that *SLC2A3* was positively related to stromal, immune, and ESTIMATE score associated with tumor invasion.

However, we used diverse online databases and R software to cross-validate the results systematically in our study, but several issues remain to be addressed. First, it would be of value to perform *in vitro/in vivo* studies to verify these results. Second, an in-depth analysis of the molecular mechanism of *SLC2A3* in HNSCC is required. Moreover, further research is deserved for the lack of direct evidence of the association between *SLC2A3* and immune regulation in HNSCC.

## 5. Conclusion

In summary, our findings indicated an intimate connection between *SLC2A3* expression and HNSCC. We uncovered a role for *SLC2A3* as a potential prognostic marker and immune therapeutic target in HNSCC via bioinformatics methods for the first time, and it suggested avenues for the precise treatment of patients with HNSCC.

## Figures and Tables

**Table 1:** Clinical characteristics of head and neck squamous carcinoma (TCGA).

Characteristic	Low expression of *SLC2A3*	High expression of *SLC2A3*	*p*
*n*	251	251	
T stage, *n* (%)			0.034
T1	23 (4.7%)	10 (2.1%)	
T2	77 (15.8%)	67 (13.8%)	
T3	60 (12.3%)	71 (14.6%)	
T4	80 (16.4%)	99 (20.3%)	
N stage, *n* (%)			0.053
N0	129 (26.9%)	110 (22.9%)	
N1	41 (8.5%)	39 (8.1%)	
N2	63 (13.1%)	91 (19%)	
N3	2 (0.4%)	5 (1%)	
M stage, *n* (%)			0.029
M0	231 (48.4%)	241 (50.5%)	
M1	5 (1%)	0 (0%)	
Clinical stage, *n* (%)			0.016
Stage I	13 (2.7%)	6 (1.2%)	
Stage II	51 (10.5%)	44 (9%)	
Stage III	59 (12.1%)	43 (8.8%)	
Stage IV	118 (24.2%)	154 (31.6%)	
Radiation therapy, *n* (%)			0.823
No	78 (17.7%)	76 (17.2%)	
Yes	150 (34%)	137 (31.1%)	
Gender, *n* (%)			0.267
Female	73 (14.5%)	61 (12.2%)	
Male	178 (35.5%)	190 (37.8%)	
Age, *n* (%)			0.098
≤60	132 (26.3%)	113 (22.6%)	
>60	118 (23.6%)	138 (27.5%)	
Histologic grade, *n* (%)			< 0.001
G1	44 (9.1%)	18 (3.7%)	
G2	149 (30.8%)	151 (31.3%)	
G3	48 (9.9%)	71 (14.7%)	
G4	2 (0.4%)	0 (0%)	
Smoker, *n* (%)			0.213
No	62 (12.6%)	49 (10%)	
Yes	185 (37.6%)	196 (39.8%)	
Alcohol history, *n* (%)			0.305
No	84 (17.1%)	74 (15.1%)	
Yes	159 (32.4%)	174 (35.4%)	
Lymphovascular invasion, *n* (%)			0.567
No	109 (32%)	110 (32.3%)	
Yes	56 (16.4%)	66 (19.4%)	
Primary therapy outcome, *n* (%)			0.181
PD	25 (6%)	16 (3.8%)	
SD	4 (1%)	2 (0.5%)	
PR	1 (0.2%)	5 (1.2%)	
CR	187 (44.7%)	178 (42.6%)	
Age, meidan (IQR)	60 (53, 68)	62 (53, 69)	0.141

## Data Availability

The data analyzed in this study are available in the public online website have been mentioned in this paper.
